# Pediatric urinary tract infections caused by poultry-associated *Escherichia coli*

**DOI:** 10.1128/spectrum.03415-23

**Published:** 2024-06-12

**Authors:** Maliha Aziz, Gregg S. Davis, Daniel E. Park, Azza H. Idris, Sanjeev Sariya, Yashan Wang, Sarah Zerbonne, Lora Nordstrom, Brett Weaver, Sally Statham, Timothy J. Johnson, Joseph Campos, Eduardo Castro-Nallar, Keith A. Crandall, Zhenke Wu, Cindy M. Liu, Roberta L. DeBiasi, Lance B. Price

**Affiliations:** 1 Department of Environmental and Occupational Health, Antibiotic Resistance Action Center, George Washington University, Washington, DC, USA; 2 Milken Institute School of Public Health, George Washington University, Washington, DC, USA; 3 Division of Pediatric Infectious Diseases, Children’s National Health System, Washington, DC, USA; 4 Vaccine Research Center, National Institute of Allergy and Infectious Diseases, National Institutes of Health, Bethesda, Maryland, USA; 5 Translational Genomics Research Institute, Flagstaff, Arizona, USA; 6 Department of Veterinary and Biomedical Sciences, College of Veterinary Medicine, University of Minnesota, St. Paul, Minnesota, USA; 7 Departamento de Microbiología, Facultad de Ciencias de la Salud, Universidad de Talca, Talca, Chile; 8 Centro de Ecología Integrativa, Universidad de Talca, Talca, Chile; 9 Department of Biostatistics, University of Michigan School of Public Health, Ann Arbor, Michigan, USA; 10 Department of Pediatrics and Microbiology, Immunology and Tropical Medicine, George Washington University School of Medicine and Health Sciences, Washington, DC, USA; Instituto de Higiene Bacteriología y Virología, Montevideo, Uruguay

**Keywords:** poultry, pediatric, urinary tract infection, foodborne, *Escherichia coli*, Bayesian latent class model

## Abstract

*Escherichia coli* is the leading cause of urinary tract infections (UTIs) in children and adults. The gastrointestinal tract is the primary reservoir of uropathogenic *E. coli*, which can be acquired from a variety of environmental exposures, including retail meat. In the current study, we used a novel statistical-genomic approach to estimate the proportion of pediatric UTIs caused by foodborne zoonotic *E. coli* strains. *E. coli* urine isolates were collected from DC residents aged 2 months to 17 years from the Children’s National Medical Center Laboratory, 2013–2014. During the same period, *E. coli* isolates were collected from retail poultry products purchased from 15 sites throughout DC. A total of 52 urine and 56 poultry isolates underwent whole-genome sequencing, core genome phylogenetic analysis, and host-origin prediction by a Bayesian latent class model that incorporated data on the presence of mobile genetic elements (MGEs) among *E. coli* isolates from multiple vertebrate hosts. A total of 56 multilocus sequence types were identified among the isolates. Five sequence types—ST10, ST38, ST69, ST117, and ST131—were observed among both urine and poultry isolates. Using the Bayesian latent class model, we estimated that 19% (10/52) of the clinical *E. coli* isolates in our population were foodborne zoonotic strains. These data suggest that a substantial portion of pediatric UTIs in the Washington DC region may be caused by *E. coli* strains originating in food animals and likely transmitted via contaminated poultry meat.

**IMPORTANCE:**

*Escherichia coli* UTIs are a heavy public health burden and can have long-term negative health consequences for pediatric patients. *E. coli* has an extremely broad host range, including humans, chickens, turkeys, pigs, and cattle. *E. coli* derived from food animals is a frequent contaminant of retail meat products, but little is known about the risk these strains pose to pediatric populations. Quantifying the proportion of pediatric UTIs caused by food-animal-derived *E. coli*, characterizing the highest-risk strains, and identifying their primary reservoir species could inform novel intervention strategies to reduce UTI burden in this vulnerable population. Our results suggest that retail poultry meat may be an important vehicle for pediatric exposure to zoonotic *E. coli* strains capable of causing UTIs. Vaccinating poultry against the highest-risk strains could potentially reduce poultry colonization, poultry meat contamination, and downstream pediatric infections.

## INTRODUCTION

Urinary tract infections (UTIs) are among the most common bacterial infections in childhood. Pediatric UTI affects between 2.5% and 5% of children annually, accounting for over 1 million office visits, 500,000 emergency department visits, and 50,000 hospital admissions in the United States annually (1–6). Importantly, pediatric patients are at risk for long-term sequelae following UTIs, including renal scarring and progressive renal disease, with lifelong deleterious effects on health (7, 8). *Escherichia coli* is the most common uropathogen in pediatric populations, accounting for 70–90% of first UTIs (4, 6, 9). Translocation of uropathogenic *E. coli* from the gastrointestinal (GI) tract of the affected individual via the fecal-perineal-urethral pathway is considered the dominant route of infection (10–13). The *E. coli* strains colonizing the GI can range from benign colonizers to extraintestinal pathogenic strains. The GI tract colonization process is shaped by a combination of host characteristics and environmental exposures (14, 15) including close household contacts, companion animals, and food (10, 12, 16, 17).

Retail poultry has been identified as a potential reservoir of *E. coli* capable of causing extraintestinal infections in humans. In adults, poultry consumption is a risk factor for UTIs (18, 19) and genetic analyses have demonstrated similarities between *E. coli* contaminating retail meats and those causing human UTIs (20–22). However, it is infeasible to sufficiently sample the roughly 9 billion poultry raised for meat annually in the United States to define the *E. coli* populations colonizing these animals or to recognize poultry-to-human spillover infections using core-genome phylogenetic methods (23).

*E. coli* strains appear to acquire and shed host-adaptive genes located on mobile genetic elements (MGEs) as they transition between vertebrate hosts (23, 24). Previously, we developed a Bayesian latent class model (BLCM) to use the presence and absence of 17 source-associated MGEs to predict the origins of *E. coli* isolates in a predominantly adult population in Flagstaff, Arizona (23). In the current study, we used this model to quantify the burden of pediatric UTIs caused by the spillover of foodborne zoonotic *E. coli* (FZEC) in Washington DC.

## RESULTS

Clinical *E. coli* isolates were collected from 52 Washington DC residents aged 2 months to 17 years, including 12 infants (0–12 months), 16 toddlers (1–3 years), 11 young children (4–8 years), and 13 adolescents (10–17 years). Most patients were female (82.7%, *n* = 43/52). Fever was reported as the most common indication for urine culture among infants (*n* = 11/12) and was also common among toddlers (*n* = 6/16). Dysuria and other voiding-related symptoms, with or without abdominal/flank pain/hematuria, were the most common indications among toddlers (*n* = 10/16), young children (*n* = 7/11), and adolescents (*n* = 11/13).

To characterize *E. coli* populations circulating among retail poultry products in Washington DC, we sampled all available brands of poultry from 15 grocery stores located throughout all wards (i.e., administrative divisions). We purchased and processed 94 raw chicken and 45 raw turkey products. We recovered 106 *E. coli* isolates (67 chicken and 39 turkey isolates) from 56 unique poultry samples. One *E. coli* isolate per positive poultry sample (*n* = 56) was arbitrarily selected for sequencing.

**Fig 1:**
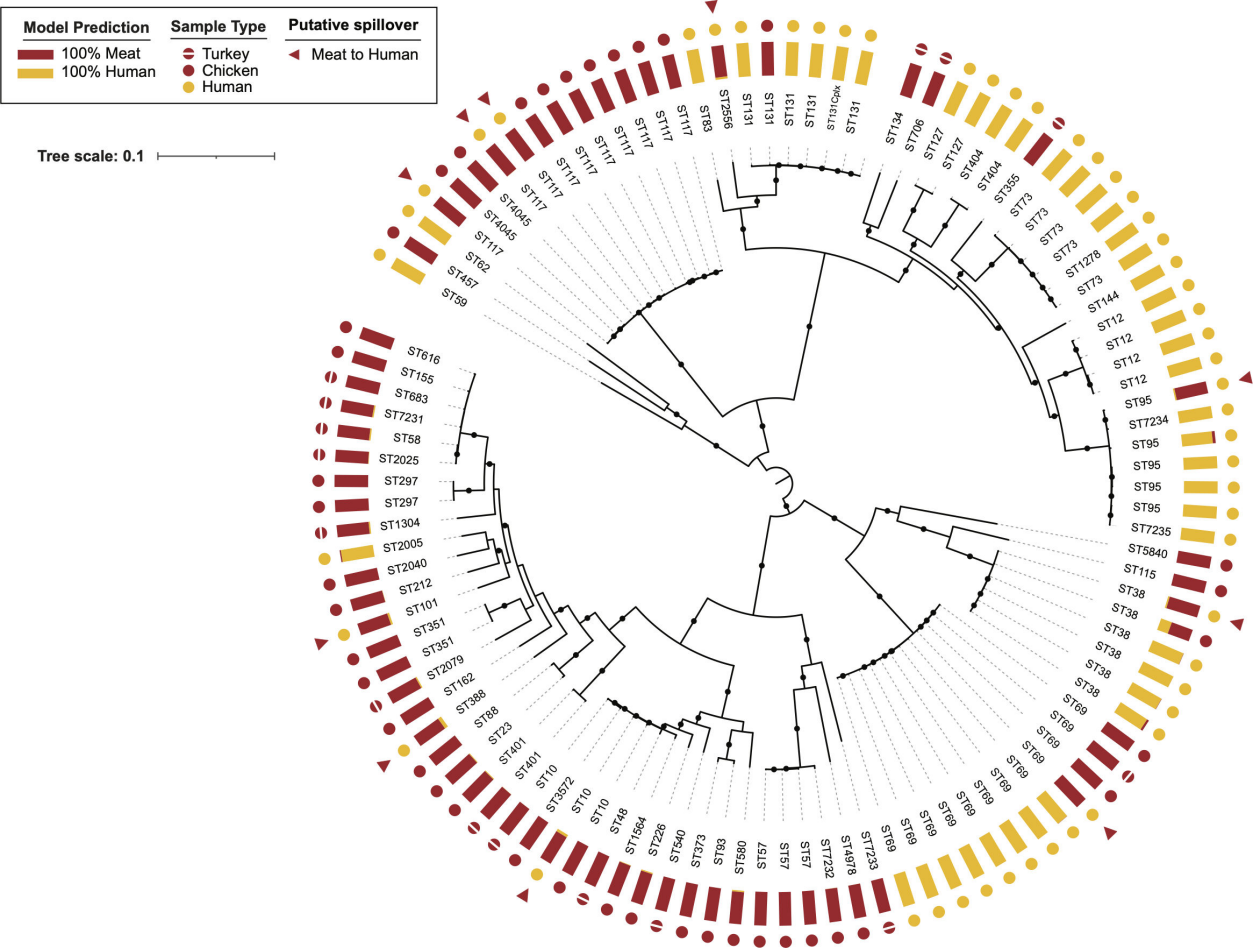
Rooted maximum-likelihood phylogeny for 108 *E. coli* isolates. The inferred phylogeny is based on 224,315 non-recombinant core-genome single nucleotide polymorphisms. Isolates are coded by multilocus sequence type (ST), sample type, and model prediction (i.e., inferred source). Clinical isolates with ≥80% probability of being from a meat source are marked by red triangles, indicating putative spillover. The scale bar reflects genetic distance, reported as the expected number of nucleotide changes per site. Black dots on branches represent bootstrap values of ≥80 (based on 100 replicates). Note: There were two ST58 isolates—recovered from duplicate packages of ground turkey—that were 100% identical at the nucleotide level; therefore, only one of the isolates was included in the phylogenetic analysis.

We identified 56 multilocus sequence typing (MLST) sequence types (STs) among all the sequenced isolates (Fig. 1 – phylogenetic tree; Table S1). We found nearly two times as many STs from the poultry isolates than from urine isolates (40 vs 22 STs; *P* < 0.0001). Five of these sequence types—ST10, ST38, ST69, ST117, and ST131—were identified among both poultry and human isolates. Twenty-three clinical urine isolates (44.2%, *n* = 23/52) and 14 poultry isolates (25.0%, *n* = 14/56) belonged to these shared sequence types.

The Bayesian latent class model predicted that 10 of the 52 clinical urine isolates (19%) were of meat origin (probability ≥0.8); whereas the model predicted that all 56 poultry *E. coli* isolates were of meat origin (Table S2). Six of the 10 clinical isolates predicted to be of meat origin were from the five shared STs, including ST10 (*n* = 1), ST117 (*n* = 3), ST38 (*n* = 1), and ST69 (*n* = 1). Our model predicted four other clinical *E. coli* isolates to be of meat origin, despite belonging to sequence types not observed among the poultry isolates sampled here. These sequence types—ST101 (*n* = 1), ST2556 (*n* = 1), ST388 (*n* = 1), and ST95 (*n* = 1)—have been observed among meat isolates in previous studies (23).

The patients infected by strains of putative meat origin were all female and represented each of the pediatric age groups, including infants (*n* = 2/12), toddlers (*n* = 1/16), young children (*n* = 4/11), and adolescents (*n* = 3/13).

We compared the resistance gene profiles of FZEC, non-FZEC, and meat isolates. FZEC isolates tended to carry fewer genes conferring resistance to medically important antibiotics as compared to non-FZEC or meat isolates (Table S3). FZEC and meat isolates were numerically more likely to carry tetracycline-resistance genes as compared to non-FZEC clinical isolates, but this difference was not statistically significant.

## DISCUSSION

Our analysis, using a novel Bayesian latent class model, suggests that over 19% of the pediatric UTIs in this study were caused by *E. coli* strains with a high likelihood of originating from meat. This was more than twice the spillover rate of FZEC previously estimated for a population consisting primarily of American adults (23). None of the poultry isolates were predicted to be of human origin.

The analytical approach that we used in this study is a powerful tool for recognizing zoonotic *E. coli* strains among extraintestinal infections in pediatric and adult populations. Core-genome phylogenetic analysis has become a cornerstone of outbreak investigations; however, it has limitations when it comes to recognizing sporadic host spillover events, especially for an organism as diverse as *E. coli*. This is further compounded by the enormity of food animal populations that may serve as reservoirs for zoonotic strains, which makes it practically impossible to sample the food supply sufficiently to define transmission patterns. By moving beyond the core genome to identify mobile genetic elements that are differentially associated with humans and the dominant food-animal species (or meat types), we have developed an approach that estimates the probability that an isolate originated from a particular source. The results for this Washington DC-based pediatric population represent the successful extension of the BLCM from our prior analysis to a geographically, demographically, and temporally distinct population. As we expand our panel of host-associated mobile genetic elements and reference genomes, we anticipate creating a tool that can be used by the infectious diseases community to quantify the burden of zoonotic *E. coli* strains to the burden of extraintestinal infections worldwide.

The study’s primary limitation was its small sample size, which made it difficult to make quantitative statements about the foodborne zoonotic *E. coli* strains that pose the greatest risk to pediatric populations. Despite this limitation, one sequence type, ST117, was found to be the most common sequence type among putative zoonotic *E. coli* infections in this study, consistent with previous work and was the most prevalent sequence type among *E. coli* isolated from poultry products (23). However, despite its prevalence in meat, ST117 was not the most common zoonotic lineage among extraintestinal infections in adults in the previous study. Future studies will have to be conducted to determine if ST117 poses a particular risk to pediatric populations and if these infections are due to specific virulence factors associated with this lineage, due to a simple stochastic effect of being so prevalent in meat, or a combination thereof. Additionally, our study included participants from a wide age range (i.e., from 2 months to 17 years), with substantially different pathophysiology, epidemiology, and etiology of UTIs. Therefore, future studies are also needed to further investigate the impact and risk factors of foodborne zoonotic *E. coli* infection across the pediatric age span.

Our findings, and those of others (18, 25–28), suggest that raw poultry is one vehicle for the transmission of uropathogenic *E. coli* strains between food animals and humans. However, the linkage is not straightforward in that young children are unlikely to handle raw poultry. Thus, other incidental exposures, such as contaminated kitchen surfaces, hands of caregivers, cross-contaminated foods, undercooked poultry, and colonized household members, including pets, are all potential routes of transmission. Unfortunately, none of these potential intermediate sources were sampled for this study.

Our findings give further context to the potential risks associated with antimicrobial use in food-animal production. However, in this study we found that the putative FZEC clinical isolates tended to carry fewer antimicrobial resistance genes as compared to non-FZEC clinical isolates. This likely reflects the relative selective pressure that these populations have undergone. Over the past two decades, the U.S. Food and Drug Administration has established more protective guidelines for the introduction of new antimicrobials to food animal production and has restricted the use of some previously approved antimicrobials (29). Despite this progress, tetracyclines are still routinely used in U.S. food animal production, which may explain the high prevalence of tetracycline resistance genes among the FZEC and meat isolates. Further investigations are needed to assess the variability in antimicrobial susceptibility among pediatric FZEC isolates in countries with different antimicrobial policies and practices (30).

UTIs account for billions of dollars in healthcare-associated costs and can impart serious long-term sequelae, particularly among pediatric patients. Understanding the epidemiology of UTI could reveal critical control points in the pathway from environmental reservoirs to infection. Here, we demonstrate evidence that retail poultry meat can serve as a vehicle for extraintestinal pathogenic *E. coli* with the potential to cause pediatric UTI. We speculate that children are exposed to these pathogens indirectly and that transmission may be reduced by improving kitchen and hand hygiene, as well as industry measures—such as vaccination programs—to decrease uropathogenic *E. coli* populations in food animals. Future studies should be conducted to measure the rate of FZEC infections in different populations that vary in age, race, geography, socioeconomic status, and public health infrastructure (i.e., water, sanitation, and hygiene).

## MATERIALS AND METHODS

### Poultry *E. coli* isolate collection

Retail chicken and turkey products representing all available brands were purchased in duplicate from 15 grocery stores located throughout all the wards of Washington DC on 13 November 2013. Meat samples were kept on ice or refrigerated until processed no later than 1 day past the sell-by date. From each package, one whole piece of meat, or 30 g ±10% of ground products, was transferred aseptically to a stomacher bag (VWR, Radnor, PA, USA) containing 250 mL MacConkey broth (Alpha Biosciences, Baltimore, MD, USA) and incubated overnight at 44°C. Enrichment culture was plated onto HardyCHROM UTI agar (Hardy Diagnostics, Santa Maria, CA, USA), four putative *E. coli* were selected from each sample and then purified on a second HardyCHROM UTI agar plate. One *E. coli* isolate per positive sample was randomly selected for subsequent analysis.

### Clinical *E. coli* isolate collection

Non-duplicate *E. coli* urine isolates were collected from DC residents aged 2 months to 17 years between October 2013 and March 2014 at the Children’s National Hospital Clinical Laboratory using standard techniques. Isolates were confirmed as *E. coli* by whole-genome DNA sequence analysis and stored at −80°C in *Brucella* broth with 20% glycerol.

### DNA sequencing and multilocus sequence typing

DNA sequencing was performed as previously described (31). We performed *de novo* assembly on Illumina paired-end reads using SPAdes (v.3.5.0) (32) and assessed the quality of the assembled scaffolds using QUAST (v.2.3) (33). MLST STs were assigned using a 7-loci MLST scheme (34). Briefly, allele numbers were identified, and STs assigned by comparing assembled genomes to all *E. coli* MLST alleles downloaded from Enterobase (35) using BLAST (v.2.2.30+) (36) with cutoff values of 100% nucleotide identity and 100% query coverage. Novel MLST alleles and STs were assigned using Enterobase.

### *In silico* antimicrobial resistance gene typing

We identified resistance determinants in the assembled genomes by screening them against Resfinder and PointFinder databases using ResFinder (v4.4.2) (37) and the following settings: species, *E. coli*; minimum coverage, 0.6; and threshold, 0.8. The resistance phenotype was determined by the presence of one or more resistance determinants per antimicrobial class.

### Core genome-based phylogenetic analyses

Single nucleotide polymorphisms were identified as previously described (31). Recombinant regions were identified and removed from the SNP matrix using Gubbins (v2.1) (38). The phylogenetic tree was rooted using a two-step process. First, an outgroup-rooted phylogeny was inferred and the placement of the root was identified. A new SNP matrix was then generated using only the ingroup isolates and the root was placed manually. We constructed a core-genome maximum-likelihood phylogeny with PhyML (39) using a general-time-reversible model of sequence evolution (40, 41), with *E. fergusonii* (GenBank accession no. CP014316) as the outgroup and *E. coli* JJ1886 (GenBank accession no. CP014316) as the reference. We calculated the support values by bootstrap sampling (*n* = 100).

### Host-origin prediction

We screened 108 isolates for 17 source-associated MGEs using minimap2 (42). We applied a BLCM that incorporates the presence or absence of these 17 MGEs to predict the host origins of clinical *E. coli* isolates, using methods described previously (43). Briefly, the Bayesian model estimates a host-origin probability for each isolate by inferring latent classes of either human or meat, using multivariate binary responses from the full set of MGEs, along with clade information from the pan-ST core genome phylogeny. Host-origin probabilities are generated using independent logistic normal priors with mean 0 and standard deviations of 1.5, while class probabilities use a Beta (1, 1) prior. User-defined posterior probability thresholds were applied for being positive (≥0.80) or negative (≤0.20) for a given source (i.e., meat vs human). Probability scores between these two values were considered indeterminate. Clinical isolates predicted to be of meat origin were identified as FZEC.

All computational analyses were performed on the George Washington University high-performance computing cluster.

## Data Availability

The sequences generated from the 108 isolates described in this study are available in the NCBI short read archive (SRA) under the accession number PRJNA485696.

## SUPPLEMENTAL MATERIAL

Table S1 spectrum.03415-23-s0001.xlsx Isolate descriptions.

Table S2 spectrum.03415-23-s0002.xlsx BLCM source predictions.

Table S3 spectrum.03415-23-s0003.xlsx Resistance gene profiles of FZEC, non-FZEC, and meat isolates.

ASM does not own the copyrights to Supplemental Material that may be linked to, or accessed through, an article. The authors have granted ASM a non-exclusive, world-wide license to publish the Supplemental Material files. Please contact the corresponding author directly for reuse.
